# Post-infectious cough in patients with obesity: an observational study

**DOI:** 10.3389/fmed.2025.1460929

**Published:** 2025-07-10

**Authors:** Yin Xian, Yuan Zhang, Xiangxin Kong, Ke Song

**Affiliations:** ^1^Department of Otorhinolaryngology, Nanchong Psychosomatic Hospital, Nanchong, China; ^2^Department of Gastroenterology, Affiliated Hospital of North Sichuan Medical College, Nanchong, China

**Keywords:** post-infectious cough, upper respiratory tract infection, insulin resistance, obesity, interaction

## Abstract

**Background:**

Upper respiratory tract infection (URTI) can lead to post-infectious cough (PIC). It is currently unclear the current status and influencing factors of PIC in patients with obesity.

**Methods:**

Data were collected from patients who visited the institution’s bariatric surgery clinic, between June and November 2022, but did not undergo surgery. Follow-up calls will be conducted in early January 2023, and whether these patients develop URTI and cough in December 2022 will be recorded. For patients with URTI and cough, the occurrence of accompanying symptoms during the course of the disease will be recorded. Then, in late February 2023, we will assess whether the cough or other accompanying symptoms have completely resolved within 8 weeks. Logistic regression analyzed the influencing factors of URTI developing into PIC in obese patients. Subgroup analyzes were used to assess interaction effects.

**Results:**

Out of 286 study participants, 54 (18.9%) had a PIC. Waist circumference (WC) [odds ratio (OR) 1.07, 95% confidence interval (CI): 1.03–1.12], homeostasis model assessment for insulin resistance (OR 1.51, 95% CI: 1.15–1.98), and vomiting during the acute phase (OR 3.42, 95% CI: 1.39–8.4) were associated with PIC in patients with obesity. Vomiting during the acute phase (p for interaction = 0.033) can affect the risk of WC to PIC.

**Conclusion:**

WC, insulin resistance, and vomiting during the acute phase are risk factors for PIC in patients with obesity. Reducing visceral fat and increasing insulin sensitivity in obese patients may help alleviate the burden of PIC.

## 1 Introduction

Post-infectious cough (PIC) is a cough that continues after the acute phase symptoms of an upper respiratory tract infection (URTI) have resolved, typically lasting 3–8 weeks. PIC commonly occurs following infections caused by various respiratory viruses, including rhinovirus, respiratory syncytial virus (RSV), coronavirus, influenza virus, and parainfluenza virus (1–4). Around 11 to 25% of patients develop PIC after URTI, and the incidence of PIC increases during influenza (5, 6). This persistent coughing can spread virus-containing droplets, increasing transmission risk, and causing psychological distress. Effective treatment options for the disease remain limited (7–9). The ongoing OSPIC trial is currently investigating the impact of oral glucocorticoids on the treatment of PIC (10). However, the use of oral corticosteroids in special populations, such as those with diabetes, hypertension, osteoporosis, and other chronic systemic diseases, may be limited due to contraindications. These diseases are all related to obesity (11–14).

Over the recent decades, global obesity prevalence has tripled. In 2016, 13% of adults were classified as obese, and 39% overweight (15), the quantities are 650 million and 1.9 billion, respectively.

As mentioned earlier, there is currently a scarcity of effective treatment options for PIC in patients with obesity and associated complications. Thus, focusing on the factors that influence the progression from URTI to PIC in these patients can establish a theoretical foundation for alleviating the burden of PIC. However, there is a lack of research on PIC in patients with obesity.

On December 7, 2022, mainland China’s abandonment of its zero-COVID-19 policy led to a peak in omicron variant infections and its clinical manifestations were mainly URTI (16, 17). A subset of obese patients opted not to undergo surgery following their attendance at the bariatric surgery clinic within this research institution. The comprehensive clinical and laboratory data presented a unique opportunity to study the occurrence and influencing factors of PIC in patients with obesity. To minimize potential changes in their health status, the study enrolled individuals who received no further treatment or no change in original treatment between June and November 2022. We monitored the occurrence of cough and post-infectious cough in these patients in December 2022, and analyzed the factors influencing the development of PIC.

## 2 Patients and methods

### 2.1 Process of research

Clinical data and laboratory results were retrospectively collected from obese patients who visited the bariatric surgery clinic at the Affiliated Hospital of North Sichuan Medical College, between June and November 2022, but did not undergo surgery. Subsequently, in early January 2023, phone interviews were conducted to inquire about any ongoing treatment for obesity and diabetes received after their clinic visits.

We gathered information regarding the URTI status of untreated patients who received no treatment or no change in original treatment. The diagnostic criteria for URTI were acute URTI symptoms accompanied by a positive polymerase chain reaction test, a self-tested antigen kit result, or exposure to confirmed COVID-19 cases. Patients diagnosed with URTI were further questioned about any coughing during their illness. For those who coughed, accompanying symptoms and chest radiological diagnosis were documented, and follow-up plans were determined based on cough duration.

- If the cough resolved within 3 weeks, it was classified as an acute cough.
- Patients who had been coughing for less than 3 weeks at the initial assessment were re-contacted after 3 weeks. If the cough had resolved, it was classified as acute; otherwise, a further follow-up was conducted in late February 2023.
- Patients who were currently experiencing cough and had a duration of more than 3 weeks were followed up in late February 2023.

In February 2023, all remaining participants were queried about cough resolution and accompanying symptoms within the previous 8 weeks. Resolved coughs were classified as subacute, and persistent coughs as chronic coughs.

PIC is defined as a cough without sputum or with a small amount of mucus-like sputum that persists for 3–8 weeks following an URTI.

### 2.2 Inclusion and exclusion criteria

The inclusion criteria were as follows: a. Patients with obesity (body mass index [BMI] ≥ 28 kg/m^2^) (18, 19) who visited the bariatric surgery clinic of North Sichuan Medical College between June and November 2022, and did not undergo surgery; b. Patients diagnosed with URTI and experiencing acute cough; c. Patients who do not meet any of the exclusion criteria listed below.

The exclusion criteria were as follows: a. Patients who underwent surgery or drug treatment for obesity and obesity-related diseases before December 2022; b. Patients with incomplete clinical and laboratory data before December 2022; c. Patients who were coughing before December 2022; d. Patients who were lost to follow-up or refused to participate during the telephone follow-up in early January 2023; e. Pregnant women.

### 2.3 Indexes collected and calculated

The collected and calculated indices based on clinical data and laboratory results obtained before December 2022. These included demographics (sex, age, weight, height, and marital status), waist circumference (WC), smoking status, blood pressure, fasting blood glucose (FBG), fasting insulin (INS), hemoglobin A1c (HbA1c), total white blood cell (WBC) count, lymphocyte count, and platelet count. Additionally, BMI was calculated as weight divided by height squared, and homeostasis model assessment for beta-cell function (HOMA2% B) and INS resistance (HOMA2-IR) were derived from FBG and INS using the University of Oxford Diabetes Trials Unit HOMA Calculator software (20).

During the follow-up in early January 2023, participants reported the presence of any accompanying symptoms, including muscle aches, sore throat, fatigue, nasal congestion, runny nose, vomiting, fever, anosmia, hypogeusia, sweating, dyspnea, and lung infection (confirmed by local chest radiography).

Diabetes based on a FBG ≥ 7.0 mmol/L or HbA1c ≥ 6.5% or using hypoglycemic drugs (21); and hypertension was defined as systolic blood pressure ≥ 130 mmHg or diastolic blood pressure ≥ 80 mmHg or taking antihypertensive drugs (22). Gout was based on previous diagnosis.

### 2.4 Instruments used in the research

INS and HbA1c levels were measured using an ADVIA Centaur XP-2 analyzer (Siemens, Germany), and blood cell counts were analyzed using a BC-6800-4 Auto Hematology Analyzer (Mindray, China).

### 2.5 Statistical analysis

All analyses were performed using R Statistical Software (Version 4.2.2,^1^ The R Foundation) and Free Statistics analysis platform (Version 1.8, Beijing, China). Continuous variables are presented as mean ± standard deviation or median and interquartile range, while categorical variables are presented as n (%). Student’s t-test or χ^2^/Fisher’s exact test was used to assess differences between the PIC and non-PIC groups. Variables with a *p* value < 0.1 in the univariate analysis were included in the subsequent multivariate binary logistic regression analysis. Subgroup analyzes were used to assess interaction effects. Statistical significance was set at *p* < 0.05.

## 3 Results

From June to November 2022, 607 individuals visited the bariatric surgery clinic at the Affiliated Hospital of North Sichuan Medical College but did not undergo surgery. Of these, 36 were excluded due to incomplete clinical or laboratory data. During the follow-up in early January 2023, 16 patients were lost to follow-up, four refused to participate, 19 underwent bariatric surgery in other hospitals, 23 received medication and 6 had their treatment plan changed. Additionally, six patients experienced coughing before December 2022. A total of 497 patients were included for further investigation.

**Figure 1:**
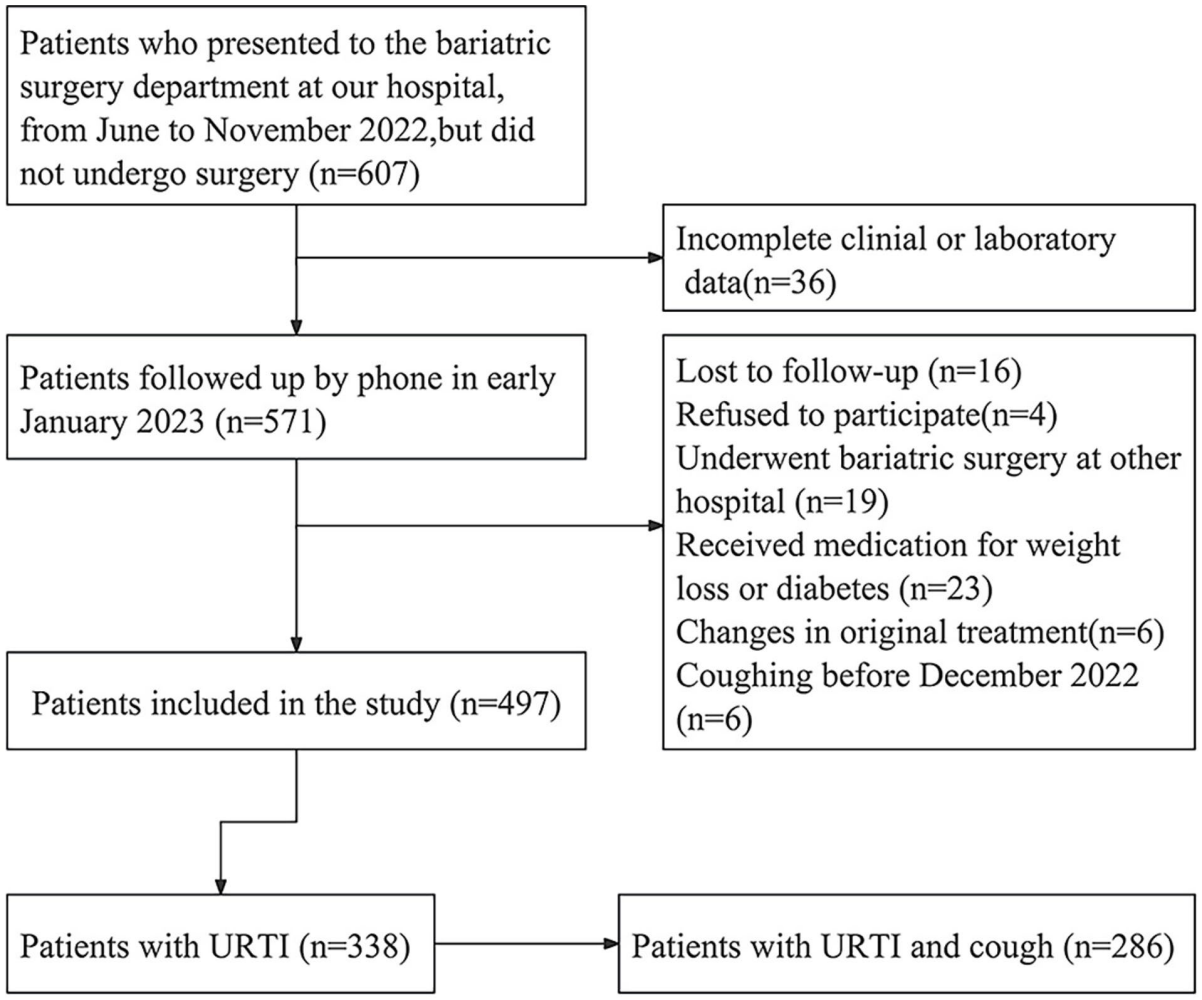
Flow chart of the selection of eligible patients.

Among these 497 patients, 338 (68.0%) reported having URTI during the January follow-up, with 286 experiencing cough symptoms (Figure 1 illustrates the process for selecting eligible patients). Among these patients, 64 had a cough for over 3 weeks. Two were diagnosed with pneumonia and three with sinusitis at the local hospital. Five had a cough for more than 8 weeks at the last follow-up. Additionally, six patients had fatigue, 10 had anosmia, four had dyspnea, and seven had hypogeusia for more than 8 weeks.

The five patients with coughs exceeding 8 weeks were advised to seek diagnosis and treatment at the local hospital’s respiratory medicine department or Otorhinolaryngology department. In the late March 2023 follow-up, one patient was diagnosed with sinusitis and experienced recovery after medical treatment. No abnormalities were detected in the upper or lower respiratory tracts of the remaining four patients. Following antitussive treatment, cough symptoms were relieved in two patients. Although symptoms improved temporarily in the other two patients, their cough recurred after discontinuing medication. During the late April 2023 follow-up, one patient was diagnosed with gastroesophageal reflux at a local hospital, and subsequent omeprazole treatment alleviated the cough. Another patient reported during the late June 2023 follow-up that antitussive drugs gradually reduced symptoms by mid-May 2023.

Among the 286 patients with acute cough, 100 were male and 186 were female, with a mean age of 34.3 (9.2) years, and a mean BMI of 37.2 (2.8) kg/m^2^. 47 (16.4%) and 159 (55.6%) of these patients had diabetes and hypertension. 54 patients were diagnosed with PIC. Table 1 presents the data for patients with acute cough.

**Table 1:** Univariate analysis of post-infectious-cough occurrence.

Variables	Total (*n* = 286)	Non-PIC (*n* = 232)	PIC (*n* = 54)	*p*	statistic
Sex, n (%)				**0.029**	**4.754**
Female	**186 (65.0)**	**144 (62.1)**	**42 (77.8)**		
Male	**100 (35.0)**	**88 (37.9)**	**12 (22.2)**		
Age group, n (%)				0.596	0.282
< 34	139 (48.6)	111 (47.8)	28 (51.9)		
≥ 34	147 (51.4)	121 (52.2)	26 (48.1)		
BMI group, n (%)				0.558	0.343
< 37	116 (40.6)	96 (41.4)	20 (37)		
≥ 37	170 (59.4)	136 (58.6)	34 (63)		
Smoke, n (%)				0.404	0.696
No	238 (83.2)	191 (82.3)	47 (87)		
Yes	48 (16.8)	41 (17.7)	7 (13)		
Married, n (%)				0.387	0.748
No	77 (26.9)	65 (28)	12 (22.2)		
Yes	209 (73.1)	167 (72)	42 (77.8)		
Diabetes, n (%)				0.202	1.624
No	239 (83.6)	197 (84.9)	42 (77.8)		
Yes	47 (16.4)	35 (15.1)	12 (22.2)		
Hypertension, n (%)				0.547	0.362
No	127 (44.4)	105 (45.3)	22 (40.7)		
Yes	159 (55.6)	127 (54.7)	32 (59.3)		
Gout, n (%)				1	Fisher
No	281 (98.3)	228 (98.3)	53 (98.1)		
Yes	5 (1.7)	4 (1.7)	1 (1.9)		
Platelet(10^6^/L)	205.2 ± 41.9	205.4 ± 42.2	204.3 ± 41.1	0.869	0.027
Fever, n (%)				0.269	1.22
No	81 (28.3)	69 (29.7)	12 (22.2)		
Yes	205 (71.7)	163 (70.3)	42 (77.8)		
Sore throat, n (%)				0.692	0.157
No	171 (59.8)	140 (60.3)	31 (57.4)		
Yes	115 (40.2)	92 (39.7)	23 (42.6)		
Nasal congestion/runny nose, n (%)				0.501	0.452
No	126 (44.1)	100 (43.1)	26 (48.1)		
Yes	160 (55.9)	132 (56.9)	28 (51.9)		
Fatigue, n (%)				0.295	1.099
No	156 (54.5)	130 (56)	26 (48.1)		
Yes	130 (45.5)	102 (44)	28 (51.9)		
Vomit, n (%)				**0.004**	**8.437**
No	**258 (90.2)**	**215 (92.7)**	**43 (79.6)**		
Yes	**28 (9.8)**	**17 (7.3)**	**11 (20.4)**		
Anosmia, n (%)				1	Fisher
No	262 (91.6)	212 (91.4)	50 (92.6)		
Yes	24 (8.4)	20 (8.6)	4 (7.4)		
Hypogeusia, n (%)				0.474	Fisher
No	274 (95.8)	221 (95.3)	53 (98.1)		
Yes	12 (4.2)	11 (4.7)	1 (1.9)		
Sweating, n (%)				0.458	0.55
No	251 (87.8)	202 (87.1)	49 (90.7)		
Yes	35 (12.2)	30 (12.9)	5 (9.3)		
Dyspnea, n (%)				1	Fisher
No	266 (93.0)	216 (93.1)	50 (92.6)		
Yes	20 (7.0)	16 (6.9)	4 (7.4)		
Pneumonia, n (%)				1	Fisher
No	88 (89.8)	72 (88.9)	16 (94.1)		
Yes	10 (10.2)	9 (11.1)	1 (5.9)		
HbA1c (%)	5.4 (5.2, 6.0)	5.4 (5.2, 5.9)	5.5 (5.2, 6.1)	0.819	0.053
BMI (kg/m^2^)	37.0 (35.0, 39.0)	37.0 (35.0, 39.0)	37.5 (36.0, 40.0)	0.189	1.722
Age (year)	34.0 (27.0, 39.8)	34.0 (27.8, 39.2)	33.0 (25.2, 39.8)	0.641	0.217
WBC (10^9^/L)	**7.2 (5.9, 8.3)**	**7.1 (5.8, 8.2)**	**7.9 (6.0, 8.8)**	**0.074**	**3.196**
Lymphocytes (10^9^/L)	**2.1 (1.8, 2.3)**	**2.1 (1.8, 2.3)**	**2.2 (2.0, 2.5)**	**0.011**	**6.496**
WC (cm)	**115.0 (109.0, 120.0)**	**114.0 (108.8, 119.0)**	**119.0 (114.0, 122.0)**	**0.002**	**9.952**
HOMA2 %B	**90.1 (77.9, 103.0)**	**88.2 (78.3, 97.8)**	**97.0 (77.9, 121.0)**	**0.044**	**4.072**
HOMA2-IR	**1.4 (1.1, 2.2)**	**1.4 (1.1, 1.9)**	**1.8 (1.3, 2.8)**	**0.017**	**5.667**

Bolded values represent variables with *p*-values less than 0.05.

### 3.1 Univariate analysis

To identify potential factors associated with PIC, univariate analysis was conducted. This analysis included demographic indicators, laboratory results, calculated indicators (all collected before December 2022), and symptoms and chest radiograph findings recorded during URTI. The results revealed that WC, HOMA2-IR, HOMA2 %B, lymphocyte count and vomiting during the acute phase were associated with a higher risk of persistent coughing. Additionally, females were found to have a higher risk than males (Table 1).

### 3.2 Multivariate analysis

Variables with *p* values less than 0.1 in the univariate analysis were included in the subsequent multivariate binary logistic regression analysis. These variables included sex, WC, HOMA2-IR, lymphocyte count, WBC count, and vomiting during the acute phase. The analysis identified WC, HOMA2-IR, and vomiting as predictors of PIC (Table 2).

**Table 2:** Multivariate analysis of PIC occurrence.

Variable	OR (95%CI)	*p* value
Sex male	0.52 (0.25–1.09)	0.084
Vomit yes	**3.42 (1.39–8.4)**	**0.007**
WBC (10^9^/L)	1.14 (0.86–1.51)	0.347
Lymphocytes(10^9^/L)	1.87 (0.63–5.52)	0.256
WC (cm)	**1.07 (1.03–1.12)**	**0.002**
HOMA2-IR	**1.51 (1.15–1.98)**	**0.003**
HOMA2 %B	0.99 (0.98–1)	0.181

Bolded values represent variables with *p*-values less than 0.05.

### 3.3 Subgroup analysis of the relationship between WC, HOMA2-IR and PIC

**Figure 2:**
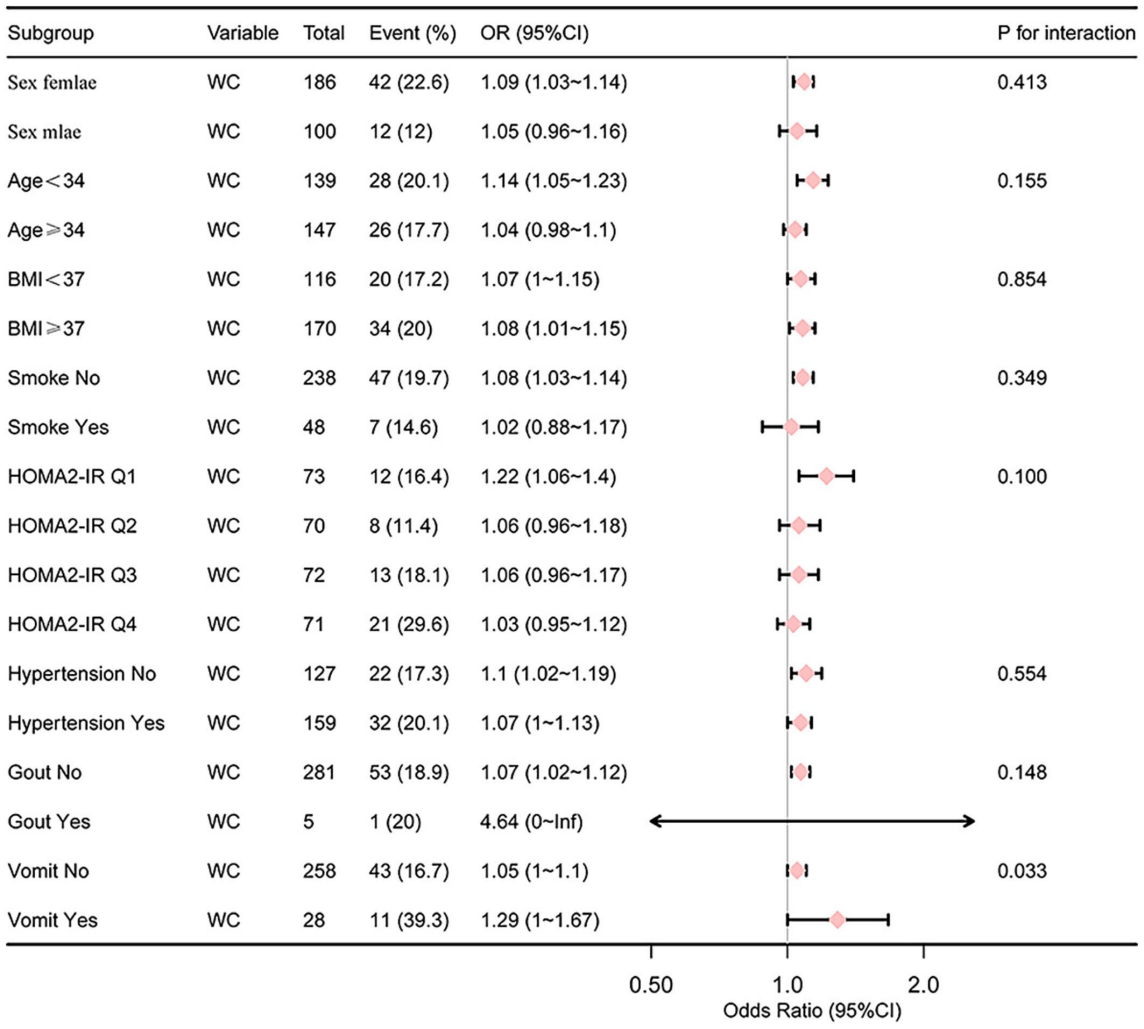
Subgroup analysis of the relationship between WC and PIC.

**Figure 3:**
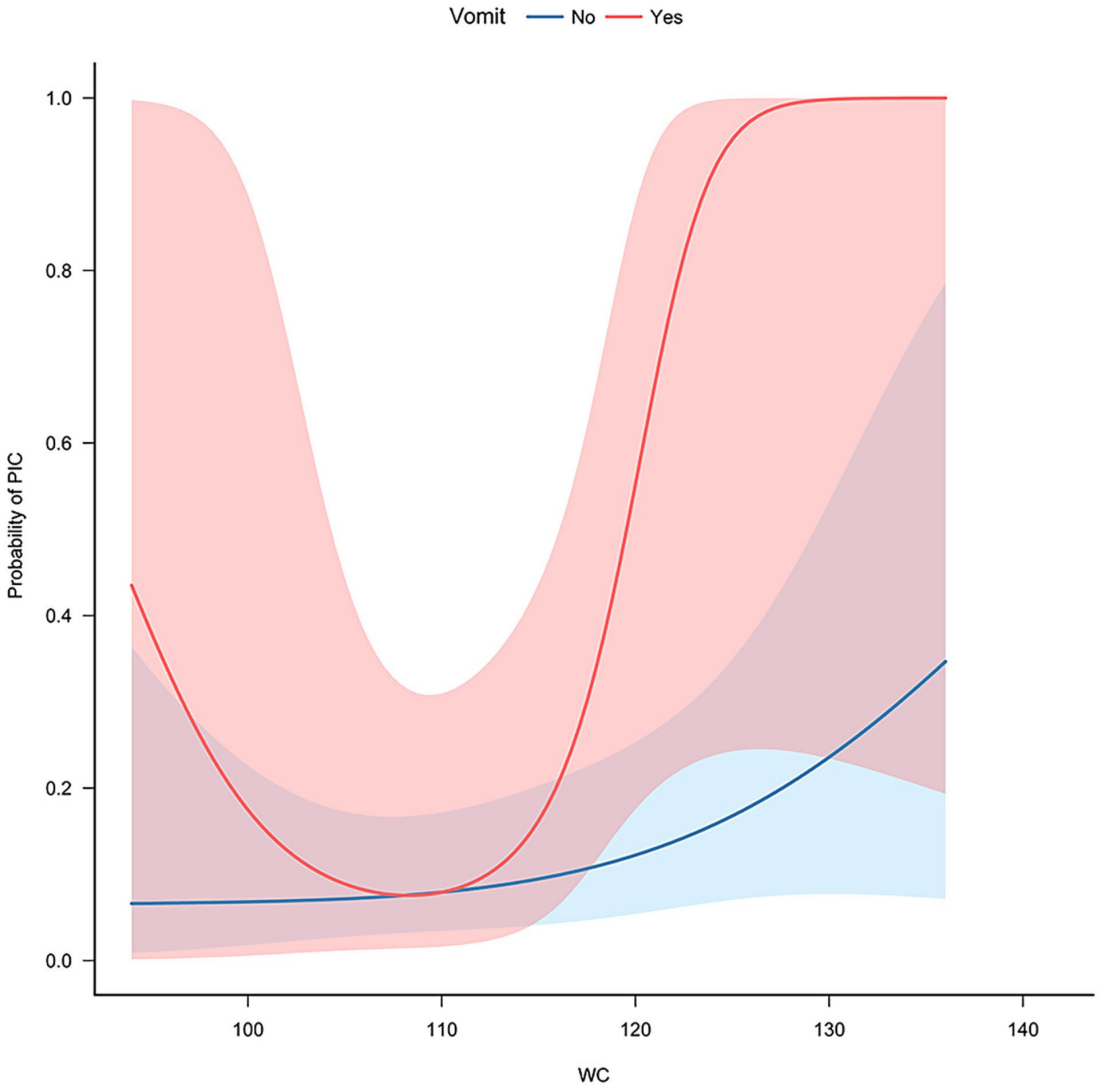
Subgroup curve fitting of WC and PIC risk based on vomiting during the acute phase.

Gender, age (<34, ≥34), BMI (<37, ≥37), diabetes (no/yes), hypertension (no/yes), acute phase vomiting (no/yes), gout (no/yes), smoke(no/yes) and quartiles of HOMA2-IR were used as stratification variables to assess the association between WC and PIC. The analysis revealed that vomiting during the acute phase (p for interaction = 0.033) have a significant impact on the risk of PIC in relation to WC (Figures 2, 3).

**Figure 4:**
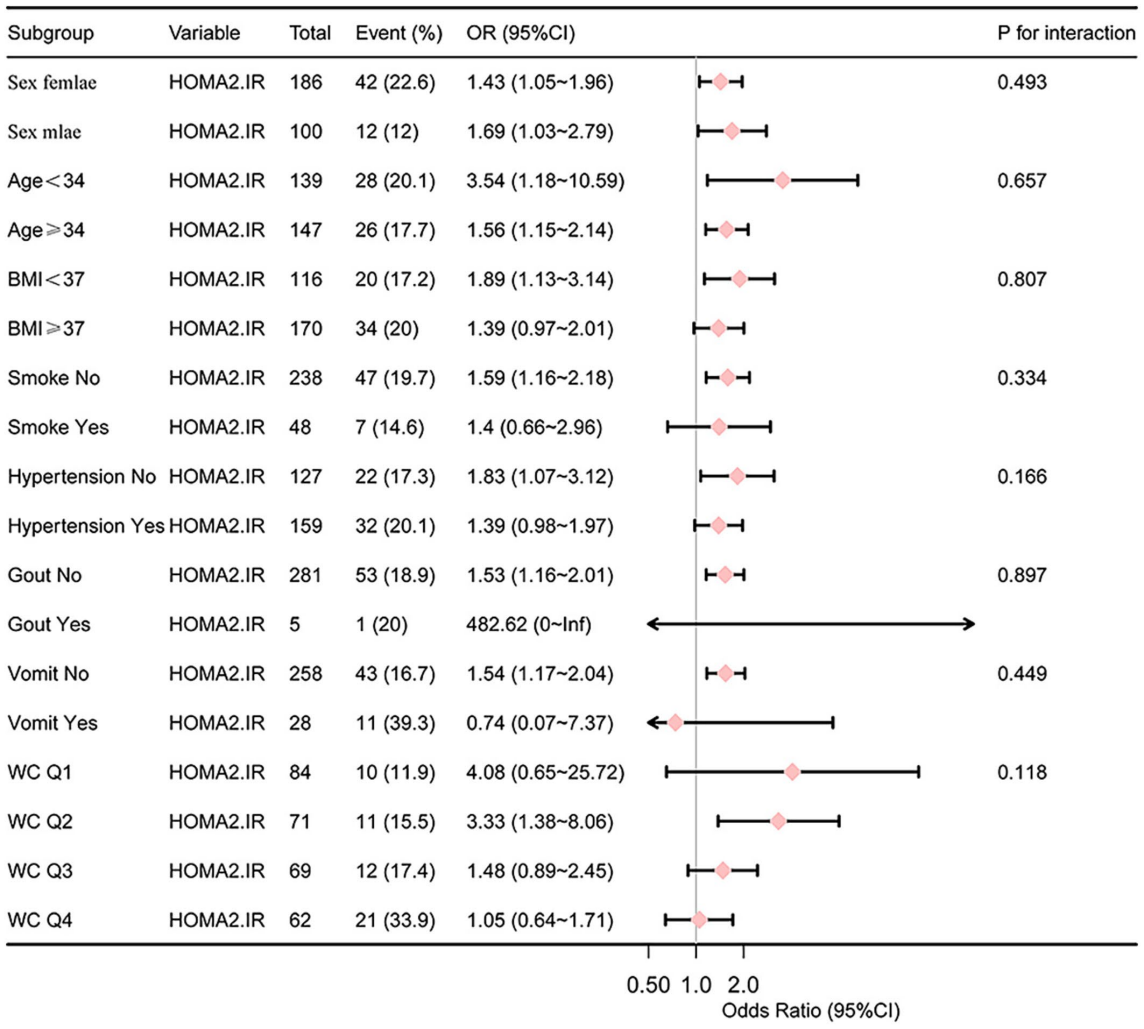
Subgroup analysis of the relationship between HOMA2-IR and PIC.

In the subgroup analysis of the relationship between HOMA2-IR and PIC, stratification variables included gender, age, BMI, diabetes, hypertension, acute phase vomiting, and quartiles of WC. The p for interaction of all variables is greater than 0.05 (Figure 4).

## 4 Discussion

This study observed that 18.9% of patients with URTI developed post-infectious cough PIC, a rate similar to the incidence of PIC in hospitalized COVID-19 patients reported in 2020 (20.9%) (23), but lower than the 43% observed in influenza A H1N1 (5). The majority of patients included in this study were infected with the Omicron variant, which reduces the impact of different pathogens on the study results. Additionally, the concentration of patient onset in December 2022 helps mitigate the influence of seasonal changes.

A previous study identified digestive symptoms during hospitalization and current smoking as risk factors for PIC related to COVID-19 (23). In a questionnaire-based study, pre-existing diabetes, asthma, and chronic cough were associated with an increased risk of persistent cough lasting more than 3 weeks (24). The present study revealed overlapping risk factors with previous findings, such as vomiting (a digestive symptom) and elevated HOMA2-IR levels, a marker of insulin resistance associated with diabetes. Notably, WC emerged as an independent risk factor for PIC progression in patients with obesity.

The finding that WC is a risk factor for PIC in obese patients aligns with the known correlation between WC and visceral fat (25–27). Previous research suggests that the involuntary cough reflex involves coordinated contraction of the chest, abdominal, and pelvic muscles. This contraction increases intra-abdominal pressure, displaces the diaphragm toward the chest cavity, and generates the force needed for airway clearance (28). In patients with abdominal obesity, the movement of visceral fat during muscle contraction might weaken the force required for airway clearance, potentially leading to the retention of secretion. Song et al. (29) proposed that PIC could be related to vagal hyperresponsiveness caused by: 1. SARS-CoV-2 infecting the sensory vagus nerve and triggering neuroinflammation through glial cells; and 2. virus stimulation of innate immune cells, leading to the release of inflammatory cytokines that activate or sensitize the vagus nerve. Therefore, we hypothesize that retention of secretions may exacerbate these responses, leading to prolonged cough duration. Recent studies have indicated that SARS-CoV-2 has the ability to infect sensory neurons present in the dorsal root ganglia, thereby sensitizing the cough reflex (30). It is important to note that the alveoli, the primary site of pulmonary infection, lack the distribution of dorsal root ganglion sensory neurons (31). Consequently, infection confined to the alveoli does not stimulate these sensory neurons. This observation may help explain why the study did not find an increased risk of PIC associated with pulmonary infection.

INS resistance is a known risk factor for metabolic diseases. Interestingly, this study also found a link between high HOMA2-IR and increased risk of PIC. Two potential mechanisms may explain this association: 1. Research suggests a two-way relationship between INS resistance and pulmonary dysfunction (32–34). In individuals with INS resistance, lung function may be compromised, potentially weakening their ability to cough effectively. This impaired clearance could contribute to persistent coughing. 2. A mouse study indicated that INS resistance, through the tumor growth factor-β1 pathway, contributes to airway hyperresponsiveness and macrophage infiltration induced by a high-fat diet (35). Similarly, when Pathogen breaches the epithelial barrier, macrophages may phagocytize the virus and release pro-inflammatory cytokines. These cytokines could stimulate the vagus nerve and amplify airway hyperresponsiveness, leading to prolonged coughing.

The analysis of the data revealed interesting findings regarding the relationship between WC, vomiting during the acute phase, and the risk of PIC. When WC exceeds approximately 110, the risk of PIC increases at a significantly higher rate in patients with vomiting compared to those without vomiting. This suggests a synergistic effect between acute phase vomiting and WC as risk factors for PIC. However, when the WC is below approximately 110, the reason why the PIC (post-infection complications) risk in vomiting patients increases as the WC decreases has not yet been explained and requires further research.

This study identified WC, HOMA2-IR, and vomiting as risk factors for the progression from URTI to PIC. These findings suggest that individuals with abdominal obesity or inadequately controlled type 2 diabetes are more susceptible to developing PIC after URTI. This highlights the need for targeted monitoring of these high-risk groups during URTI episodes. Moreover, if validated in large-scale prospective studies, these factors could serve as the foundation for a clinical prediction model to estimate the likelihood of PIC development during URTI. Such a model could enable early patient education in high-risk individuals, potentially reducing the clinical and psychosocial burden associated with the onset of PIC.

This study had several limitations: 1. the participants’ pre-infection status was determined based on data collected between June to November 2022, which could be subject to change. However, including patients who did not received further treatment or no change in original treatment during this period helps minimize the impact of potential alterations. 2. Chest radiographs were used to assess lung infections. Therefore, a single negative result might not capture the entire disease course. Further research is needed to fully understand the influence of pulmonary infections on PIC. 3. The single-center retrospective design of this study introduces inherent limitations, including potential selection bias and limited statistical power due to a small sample size. Future multicenter prospective studies are warranted to confirm and generalize these findings.

## 5 Conclusion

This study suggests that WC and INS resistance may increase the risk of PIC in obese individuals. Consequently, acute coughing in patients with abdominal obesity and INS resistance is more likely to progress to PIC. Therefore, managing abdominal obesity, diabetes, or prediabetes may hold the potential for reducing PIC risk in obese individuals. This research contributes to our understanding of URTI in obesity and ultimately may help alleviate the burden it places on public health.

## Data Availability

The raw data supporting the conclusions of this article will be made available by the authors, without undue reservation.

## Footnotes

^1^ http://www.R-project.org
